# Collision-Free Compliance Control for Redundant Manipulators: An Optimization Case

**DOI:** 10.3389/fnbot.2019.00050

**Published:** 2019-07-11

**Authors:** Xuefeng Zhou, Zhihao Xu, Shuai Li

**Affiliations:** ^1^Guangdong Key Laboratory of Modern Control Technology, Guangdong Institute of Intelligence Manufacturing, Guangzhou, China; ^2^School of Engineering, Swansea University, Swansea, United Kingdom

**Keywords:** recurrent neural network, compliance control, redundant manipulator, obstacle avoidance, zeroing neural network

## Abstract

Force control of manipulators could enhance compliance and execution capabilities, and has become a key issue in the field of robotic control. However, it is challenging for redundant manipulators, especially when there exist risks of collisions. In this paper, we propose a collision-free compliance control strategy based on recurrent neural networks. Inspired by impedance control, the position-force control task is rebuilt as a reference command of task-space velocities, by combing kinematic properties, the compliance controller is then described as an equality constraint in joint velocity level. As to collision avoidance strategy, both robot and obstacles are approximately described as two sets of key points, and the distances between those points are used to scale the feasible workspace. In order to save unnecessary energy consumption while reducing impact of possible collisions, the secondary task is chosen to minimize joint velocities. Then a RNN with provable convergence is established to solve the constraint-optimization problem in realtime. Numerical results validate the effectiveness of the proposed controller.

## 1. Introduction

Industry 4.0 is becoming a label of modern industry combining traditional manufacturing and increasingly technological world. As an important executor, robot manipulator must be more flexible and intelligent, to satisfy production requirements which is more personalized and customized (Gonzalez et al., 2018). Among various kinds of robot manipulators, redundant manipulators have become an important branch of robotics due to its flexibility (Zhang, 2015). This enables robots to fulfill more complicated tasks and has been a hot topic in the field of robotic control.

With the increasing popularity of robot manipulators, traditional position control based applications (such as welding, painting and so on) can hardly meet complex production tasks (He et al., 2015), for instance, in pure position control based structures, the interaction between robot and workpieces is usually ignored, which could probably lead to high security risk, since excessive system stiffness would lead to the unpredictable responses (Cai and Xiang, 2018). Therefore, aiming at enhancing the execution ability of the system, precise control of contact force is required to ensure compliance to external environment. Accordingly a series of control methods are proposed, depending on different robotic structure and control signals. By imitating flexible joints and muscles of animals, compliance units are introduced into the robots, such as series elastic actuators (SEA), variable stiffness actuators, etc. In Pan et al. (2018b), a compliance controller is designed for SEA based systems, and a modified command-filtered back-stepping control strategy (CFBC) based on adaptive mechanism is then proposed to overcome the discontinuous friction and complexity problem of traditional back-stepping based methods. By adjusting the compliance of joint angles, precise control of torque output is realized. As to the interaction between the robot and workpieces, Hogan proposes a basic idea of impedance control, in which the robot and environment usually bear as an impedance and admittance, respectively (Hogan, 1985). Generally speaking, the contact force and relative movement of the robot and workpieces can be described as a combination of mass-spring-damper systems. Therefore, the contact force can be controlled by designing motion commands indirectly. Another representative approach is hybrid position-force control, the controller is usually designed in the torque loop of the joint space, in which both contact forces and movement of the robot are modeled based on dynamic analysis. Then the controller can be described as a combination of control efforts which achieve position and force control, respectively (Raibert and Craig, 1981). Similar research can be found in literature such as (Khatib, 1987; Pan et al., 2018a, 2019; Zhao et al., 2018a,b).

During the operating process, since the manipulators are usually required to keep in touch with the workpieces, it is possible that the robot would collide with the environment. Besides, the workspace of a robot as also limited (Khatib, 1986). For example, in a production line with multiple manipulators, each robot is located at a fixed position, in order to avoid interference, the robot's workspace is limited by hardware (fences, barriers, etc.) or software constraints(pre-planned space). In situations such as human-machine collaboration, the robot must not collide with human. Therefore, it is crucial to avoid obstacles during the operating process. In present reports, the desired trajectory is generally obtained by off-line programming, which is limited by programming efficiency. To realize obstacle avoidance control in realtime, artificial potential field based methods are widely used. The basic idea of is that the target bears as an attractive pole while the obstacle creates repulsion on the robot, then the robot will be controlled to converge to the target without colliding with obstacles (Wang et al., 2018). In Csiszar et al. (2011), a modified method is proposed, which describes the obstacles by different geometrical forms, both theoretical conduction and experimental tests validate the proposed method. Considering the local minimum problem that may caused by multi-link structures, in Badawy (2016), a two minima is introduced to construct potential field, such that a dual attraction between links enables faster maneuvers comparing with traditional methods. Other improvements to artificial potential field method can be found in Tsai et al. (2001), Tsuji et al. (2002). A series of pseudo-inverse methods are constructed for redundant manipulators in Sciavicco and Siciliano (1988), in which the control efforts consists of a minimum-norm particular solution and homogeneous solutions, and the collision can be avoided by calculating a escape velocity as homogeneous solutions. By understanding the limited workspace, the obstacle avoidance can be described in forms of inequalities, which opens a new way in realtime collision avoidance. In Zhang and Wang (2004), the robot is regarded as the sum of several links, and the distances between the robot and obstacle is obtained by calculating distances between points and links. Then Guo and Zhang (2012) improves the method by modifying obstacle avoidance MVN scheme, and simulation results show that the modified control strategy can suppress the discontinuity of angular velocities effectively.

In terms with compliance control problem of a robot, the controller efforts should be designed according to the desired commands and system characteristics. That is so say, the robot must follow a constraint that achieves compliance control, and at the same time, the inequality constraints are ensured to avoid obstacles. It is obvious that the control problem involves several constraints, including equality constraints and inequality ones. Using the thought of constraint-optimization, the control problem with multiple constraints can be well handled. Recently, the applications of recurrent neural networks for robotic control have been studied extensively, and have shown great efficient for real-time processing (Wang et al., 2015; Jin et al., 2017; Xu et al., 2019a). In those literatures, analysis in dual space and a convex projection are introduced to handle inequality constraints.

Recently, taking advantage of parallel computing, neural networks are used to solve the constraint-optimization, and have shown great efficiency in real-time processing. In Zhang et al. (2004), Li et al. (2017), Yang et al. (2018b), controllers are established in joint velocity/acceleration level, to fulfill kinematic tracking problem for robot manipulators. In Xu et al. (2019b), tracking problem with model uncertainties is considered, and an adaptive RNN based controller is proposed for a 6DOF robot Jaco_2_. Discussions on multiple robot systems, parallel manipulators, time-delay systems using RNN can be found in Zhang et al. (2018), Li et al. (2019), Xu et al. (2019b).

From the previous observations, we propose a RNN based collision-free compliance control strategy for redundant manipulators. The remainder of this paper is organized as follows. In section 2, the control objective including the position-force control as well as collision avoidance is pointed out, and then rewritten as a QP problem. In section 3, the RNN based controller is proposed, and the stability of the system is also analyzed. A number of numerical experiments on a 4-DOF redundant manipulator including model uncertainties and narrow workspace are carried out to further verify the proposed control strategy. section 5 concludes the paper. The contributions of this paper are summarized as below

- To the best of the author's knowledge, there is few research on compliance control using recurrent neural networks, the study in this paper is of great significance in enriching the theoretical frame of RNN.
- The proposed controller is capable of handling compliance control, as well as avoiding obstacles in realtime, which does make sense in industrial applications, besides, physical constraints are also guaranteed.
- Comparing to traditional neural-network-based controllers used in robotics, not only control errors but model information is considered, therefore, the proposed RNN has a simple structure, and the global convergence can be ensured.

## 2. Problem Formulation

### 2.1. Robot Kinematics and Impedance Control

Without loss of generality, we consider series robot manipulators with redundant DOFs, and the joints are assumed as rotational joints. Let θ ∈ ℝ^*n*^ be the vector of joint angles, the description of the end-effector in the cartesian space is:

(1) $$\begin{array}{r} x=f\left(\theta \right), \end{array}$$

where *x* ∈ ℝ^*m*^ is the coordination of the end-effector. In the velocity level, the forward kinematic model can be formulated as:

(2) $$\begin{array}{r} ẋ=J\left(\theta \right)\dot{\theta }, \end{array}$$

in which *J*(θ) = ∂*x*/∂θ is Jacobian matrix. As to redundant manipulators, *J* ∈ ℝ^*m* × *n*^, *rank*(*J*) < *n*.

**Figure 1:**
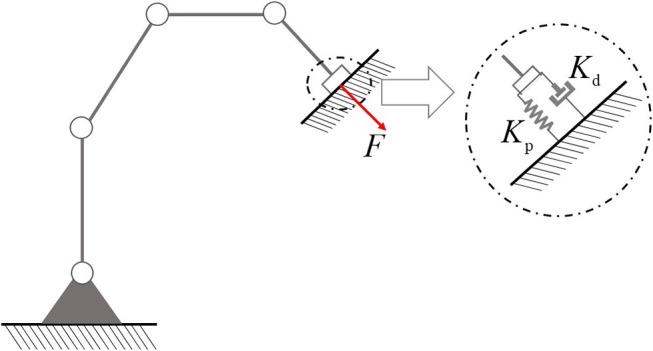
Damper-spring model of interaction between robot and workpiece.

In industrial applications, position control based operation mode has many limitations: due to the lack of compliance, pure kinematic control methods may cause unexpected consequences, especially when the robot is in contact with the environment. To enhance the compliance and achieve precise control of contact force, according to impedance control technology, the interaction between robot and environment can be described as a damper-spring system, as shown in Figure 1 (Senoo et al., 2017).

(3) $$\begin{array}{r} F=K_{p}\Delta x+K_{d}\text{d}\left(\Delta x\right)/\text{d}t, \end{array}$$

where, *K*_*p*_ and *K*_*d*_ are interaction coefficients, and Δ*x* = *x* − *x*_d_ is the difference between the actual response *x* and desired trajectory *x*_d_. The basic idea of impedance control methods is shown in Equation (2.1). By referring to Equations (2) and (3), we have:

(4) $$\begin{array}{r} ẋ=K_{d}^{-1}F-K_{p}K_{d}^{-1}\Delta x+{ẋ}_{\text{d}}. \end{array}$$

When the real values of *K*_*p*_ and *K*_*d*_ are known, *F* can be obtained by adjusting the velocity ẋ of the end-effector according to Equation (4).

### 2.2. Obstacle Avoidance Scheme

In the process of robot force control, there is a risk of collision as the robot may contact with workpieces. Besides, robot manipulators usually work in a limited workspace restricted by fences, which are used to isolated robots from humans or other robots. This problem could be even more acute in tasks which requires collaboration of multiple robots. Therefore, obstacle avoidance problem must be taken into consideration. When collision does not happens, the distance between robot and obstacles keep positive. By describing the robot and obstacles as two separated sets, namely *S*_*A*_ = {*A*_1_, ⋯ , *A*_*a*_}, *S*_*B*_ = {*B*_1_, ⋯ , *B*_*b*_}, where *A*_*i*_, *i* = 1, ⋯ , *a* and *B*_*j*_, *j* = 1, ⋯ , *b* are points on the robot and obstacles, respectively. Then the sufficient and necessary conditions of obstacle avoidance problem is that the intersection of A and B is an empty set. That is to say, for any point pair *A*_*i*_ on the robot and *B*_*j*_ on the obstacle, the distance between *A*_*i*_ and *B*_*j*_ is always positive, i.e., $||A_{i}B_{j}{||}_{2}^{2}>0$, for all *i* = 1, ⋯ , *a*, *j* = 1, ⋯ , *b*, where $||•{||}_{2}^{2}$ is the Euclidean norm of vector *A*_*i*_*B*_*j*_. For sake of safety, let *d* > 0 be a proper value describing the minimum distance between robot and obstacles, the collision can be avoided b ensuring $||A_{i}B_{j}{||}_{2}^{2}\geq d$.

*Remark 1*. In fact, both *S*_*A*_ and *S*_*B*_ consist of infinite points. However, by evenly selecting representative points from the robot link and obstacles, *S*_*A*_ and *S*_*B*_ can be simplified significantly. Besides, the safety distance *d* can be appropriately increased. Despite that this treatment will sacrifice some workspace of the robot (the inequality $||A_{i}B_{j}{||}_{2}^{2}\geq d$ would into account some areas that collisions do not happen, due to a bigger *d* is considered), this sacrifice is meaningful: the number of inequality constraints can be reduced greatly, which is helpful for constraint description and solution.

In real applications, the key points of the robot manipulator is easy to select. Cylindrical envelopes are usually used to describe the robotic links, then the key points can be selected on the axes of the cylinders uniformly, and the distance between those points can be defined the same as the radius of the cylinder. As to the obstacles with irregular shapes, the key points can be selected based on image processing techniques, such as edge detection, corrosion, etc.

### 2.3. Problem Reformulation in QP Type

From the above description, the purpose of this paper is to build a collision-free force controller for redundant manipulators, to achieve precise force control along a predefined trajectory, in the sense that *F* → *F*_d_, *x* → *x*_d_, and $||A_{i}B_{j}{||}_{2}^{2}\geq d$ for all *i* = 1, ⋯ , *a*, *j* = 1, ⋯ , *b*.

As to a redundant manipulator, there exist redundant DOFs, which can be used to enhance the flexibility of the robot. When the robot gets close to the obstacles, the robot must avoid the obstacle without affecting the contact force and tracking errors. On the other hand, when there is no risk of collision, the robot may work in an economic way, by minimizing the joint velocities, energy consumption can be reduced effectively. Therefore, by defining an objective function as $||\dot{\theta }{||}_{2}^{2}$, the control objective can be summarized as:

(5a) $$\begin{array}{rc} \text{min } & ||\dot{\theta }{||}_{2}^{2}, \end{array}$$

(5b) $$\begin{array}{rc} s.t.\; & x=x_{\text{d}}, \end{array}$$

(5c) $$\begin{array}{r} F=F_{\text{d}}, \end{array}$$

(5d) $$\begin{array}{r} ||A_{i}B_{j}{||}_{2}^{2}\geq d, \end{array}$$

where $||\dot{\theta }{||}_{2}^{2}$ is the Euclidean norm of $\dot{\theta }$. It is noteworthy that in actual industrial applications, the robot is also limited by its own physical structures. For instance, the joint angles are limited in a fixed range, and the upper/lower bounds of joint velocities are also constrained due to actuator saturation. By combing (Equation 4), the control objective rewrites to:

(6a) $$\begin{array}{rc} \text{min } & ||\dot{\theta }{||}_{2}^{2}, \end{array}$$

(6b) $$\begin{array}{rc} s.t.\; & J\dot{\theta }=K_{d}^{-1}F-K_{p}K_{d}^{-1}\Delta x+{ẋ}_{\text{d}}, \end{array}$$

(6c) $$\begin{array}{r} ||A_{i}B_{j}{||}_{2}^{2}\geq d, \end{array}$$

(6d) $$\begin{array}{r} \theta^{-}\leq \theta \leq \theta^{+}, \end{array}$$

(6e) $$\begin{array}{r} {\dot{\theta }}^{-}\leq \dot{\theta }\leq {\dot{\theta }}^{+}, \end{array}$$

with θ^−^, θ^+^, ${\dot{\theta }}^{-}$, ${\dot{\theta }}^{+}$ being the upper/lower bounds of joint angles and velocities, respectively. However, the optimization problem is described in different levels, i.e., joint speed level or joint angle level, which remains challenging to solve (Equation 6) directly. Therefore, we will rewrite this formula in velocity level. As to the key points *A*_*i*_ on the robot, let *x*_*Ai*_ be the coordination of *A*_*i*_ in the cartesian space, both *x*_*Ai*_ and ẋ_*Ai*_ are available:

(7a) $$\begin{array}{r} x_{Ai}=f_{Ai}\left(\theta \right), \end{array}$$

(7b) $$\begin{array}{r} {ẋ}_{Ai}=J_{Ai}\dot{\theta }, \end{array}$$

where *f*_*Ai*_(•) is the forward kinematics of point *A*_*i*_, and *J*_*Ai*_ is the corresponding Jacobian matrix from *A*_*i*_ to joint space. Let us consider the following equality:

(8) $$\begin{array}{r} \frac{\text{d}}{\text{d}t}\left(||A_{i}B_{j}{||}_{2}^{2}\right)=k\left(||A_{i}B_{j}{||}_{2}^{2}-d\right), \end{array}$$

in which *k* is a positive constant. It is obviously that the equilibrium point of Equation (8) is $||A_{i}B_{j}{||}_{2}^{2}=d$. By letting $\frac{\text{d}}{\text{d}t}\left(||A_{i}B_{j}{||}_{2}^{2}\right)\geq 0$, the inequality (5d) can be readily guaranteed. Taking the time-derivative of $||A_{i}B_{j}{||}_{2}^{2}$ yields:

(9) $$\begin{array}{l} \frac{\text{d}}{\text{dt}}(||B_{j}A_{i}|{|}_{2}^{2})=\frac{\text{d}}{\text{dt}}(\sqrt{{\left(A_{i}-B_{j}\right)}^{\text{T}}(A_{i}-B_{j})})\\ \;=\frac{1}{||B_{j}A_{i}|{|}_{2}^{2}}{\left(A_{i}-B_{j}\right)}^{\text{T}}\left({\dot{A}}_{i}-{\dot{B}}_{j}\right)\\ \;={\vec{\left|B_{j}A_{i}\right|}}^{\text{T}}J_{Ai}(\theta )\dot{\theta }-{\vec{\left|B_{j}A_{i}\right|}}^{\text{T}}{\dot{B}}_{j}, \end{array}$$

where, $\vec{\left|B_{j}A_{i}\right|}={\left(A_{i}-B_{j}\right)}^{\text{T}}/||\dot{\theta }{||}_{2}^{2}$ is a unit vector from *B*_*j*_ to *A*_*i*_, and Ḃ_*j*_ is the velocity of key point *B*_*j*_ on the obstacles. By Equations (9) and (6c), the inequality description of obstacle avoidance strategy is

(10) $$\begin{array}{r} {\vec{\left|B_{j}A_{i}\right|}}^{\text{T}}J_{Ai}\left(\theta \right)\dot{\theta }\geq k\left(||A_{i}B_{j}{||}_{2}^{2}-d\right)+{\vec{\left|B_{j}A_{i}\right|}}^{\text{T}}{Ḃ}_{j}, \end{array}$$

*Remark 2*. In this part, we have shown the basic idea of obstacle avoidance scheme in velocity level, whose equilibrium point is described in Equation (8). It is notable that the right-hand side of Equation (8) is only a common form to realize obstacle avoidance. Generally speaking, the right-hand side of Equation (8) may have different forms, such as $k\left(||A_{i}B_{j}{||}_{2}^{2}-d\right)$, $k{\left(||A_{i}B_{j}{||}_{2}^{2}-d\right)}^{3}$, etc. From Equation (10), the value of the response velocity to avoid obstacles is related to the two parts, the first part is the difference between the actual and safety distance, the other part depends on the movement of the obstacles.

In terms of the physical constraints of joint angles, according to escape velocity method, inequalities (6d) and (6e) can be uniformly described as $\text{max}\left(\alpha \left(\theta^{-}-\theta \right),{\dot{\theta }}^{-}\right)\leq \dot{\theta }\leq \text{min}\left({\dot{\theta }}^{+},\alpha \left(\theta^{+}-\theta \right)\right)$. So far, the position-force control problem together with obstacle avoidance strategy in velocity level is as below

(11a) $$\begin{array}{rc} \text{min } & ||\dot{\theta }{||}_{2}^{2}, \end{array}$$

(11b) $$\begin{array}{rc} s.t.\; & J\dot{\theta }=K_{d}^{-1}F-K_{p}K_{d}^{-1}\Delta x+{ẋ}_{\text{d}}, \end{array}$$

(11c) $$\begin{array}{r} \text{max}\left(\alpha \left(\theta^{-}-\theta \right),{\dot{\theta }}^{-}\right)\leq \dot{\theta }\leq \text{min}\left({\dot{\theta }}^{+},\alpha \left(\theta^{+}-\theta \right)\right), \end{array}$$

(11d) $$\begin{array}{r} J_{o}\dot{\theta }\leq B. \end{array}$$

where (11c) is a rewritten inequality considering (6d) and (6e) based on escape velocity scheme (Zhang et al., 2004), $J_{o}=\left[\underset{b}{\underset{︸}{{\vec{\left|B_{1}A_{1}\right|}}^{\text{T}}J_{A1};\cdots \;;{\vec{\left|B_{b}A_{1}\right|}}^{\text{T}}J_{Ab}}},\cdots \;,\underset{b}{\underset{︸}{{\vec{\left|B_{1}A_{a}\right|}}^{\text{T}}J_{Aa}^{\text{T}};\cdots \;;{\vec{\left|B_{b}A_{a}\right|}}^{\text{T}}J_{Ab}}}\right]$ ∈ ℝ^*ab* × *n*^ is the concatenated form of *J*_*Ai*_ considering all pairs between *A*_*i*_ and *B*_*j*_, $B={\left[B_{11},\cdots \;,B_{1b},\cdots \;,B_{a1},\cdots \;,B_{ab}\right]}^{\text{T}}\in {\mathbb{R}}^{ab}$ is the vector of upper-bounds, in which $-k\left(||A_{i}B_{j}{||}_{2}^{2}-d\right)-{\vec{\left|B_{j}A_{i}\right|}}^{\text{T}}{Ḃ}_{j}$. From the definition of *J*_*o*_, *B*, inequality (11d) in equivalent to ${\vec{\left|B_{1}A_{1}\right|}}^{\text{T}}J_{A1}\left(\theta \right)\dot{\theta }\geq k\left(||A_{1}B_{1}{||}_{2}^{2}-d\right)+{\vec{\left|B_{1}A_{1}\right|}}^{\text{T}}{Ḃ}_{1}$,…${\vec{\left|B_{b}A_{a}\right|}}^{\text{T}}J_{Aa}\left(\theta \right)\dot{\theta }\geq k\left(||A_{a}B_{b}{||}_{2}^{2}-d\right)+{\vec{\left|B_{b}A_{a}\right|}}^{\text{T}}{Ḃ}_{b}$, which is the cascading form of the inequality description (10) for all points pairs *A*_*i*_*B*_*j*_, i.e., if (11d) hold, the obstacle avoidance can be achieved. It is notable that a larger number of key points do help to describe the information of the obstacle more clearly, but it would lead to a computational burden, since the number of inequality constraints also increases. Therefore, the distance of the key points on the obstacle can be selected similar to those of the manipulator.

## 3. RNN Based Controller Design

In section II, we have transform the compliance control as well as obstacle avoidance problem into a constraint-optimization one. However, because that the QP problem described in Equation (11) contains equality and inequality constraints, moreover, both (Equations 11b,d) are nonlinear, it is difficult to solve directly, especially in industrial applications in realtime. Based on the parallel computation ability, a RNN is established to solve (Equation 11) online, and the stability of the closed-loop system is also discussed.

### 3.1. RNN Design

In terms with the QP problem (Equation 11), although the analytical solution can be hardly obtained, by defining a Lagrange function as:

(12) $$\begin{array}{r} L=||\dot{\theta }{||}_{2}^{2}+\lambda_{1}^{\text{T}}\left(K_{d}^{-1}F-K_{p}K_{d}^{-1}\Delta x+{ẋ}_{\text{d}}-J\left(\theta \right)\dot{\theta }\right)+\lambda_{2}^{\text{T}}\left(J_{o}\dot{\theta }-B\right), \end{array}$$

where λ_1_ and λ_2_ are state variables, respectively. According to Karush-Kuhn-Tucker (KKT) conditions, the inherent solution of Equation (11) satisfies:

(13a) $$\begin{array}{r} \dot{\theta }=P_{\Omega }\left(\dot{\theta }-\frac{\partial L}{\partial \dot{\theta }}\right), \end{array}$$

(13b) $$\begin{array}{r} J\dot{\theta }=K_{d}^{-1}F-K_{p}K_{d}^{-1}\Delta x+{ẋ}_{\text{d}}, \end{array}$$

(13c) $$\begin{array}{r} \lambda_{2}={\left(\lambda_{2}+J_{o}\dot{\theta }-B\right)}^{+}, \end{array}$$

where, *P*_Ω_(*x*) = argmin_*y* ∈ Ω_||*y*−*x*|| is a projection operator of $\dot{\theta }$ to convex Ω, and $\Omega =\left\{\dot{\theta }\in {\mathbb{R}}^{n}|\text{max}\left(\alpha \left(\theta^{-}-\theta \right),{\dot{\theta }}^{-}\right)\leq \dot{\theta }\leq \text{min}\left({\dot{\theta }}^{+},\alpha \left(\theta^{+}-\theta \right)\right)\right\}$. In Equation (13c), the operation function (•)^+^ is defined as a mapping to the non-negative space. Equation (13c) can be rewritten as:

(14) $$\{\begin{array}{l} \lambda_{2}>0\text{ if }J_{o}\dot{\theta }=B,\\ \lambda_{2}=0\text{ if }J_{o}\dot{\theta }\leq B, \end{array}$$

When $J_{o}\dot{\theta }\leq B$, the inequality (Equation 11d) holds, then λ_2_ stays zero. Instead, if the inequality reaches a critical state, λ_2_ becomes positive to ensure $J_{o}\dot{\theta }=B$. In order to obtain the inherent solution in real time, a recurrent neural network is built as follows:

(15a) $$\begin{array}{r} \epsilon \ddot{\theta }=-\dot{\theta }+P_{\Omega }\left(\dot{\theta }-\dot{\theta }/||\dot{\theta }{||}_{2}^{2}+J^{\text{T}}\lambda_{1}-J_{o}^{\text{T}}\lambda_{2}\right), \end{array}$$

(15b) $$\begin{array}{r} \epsilon {\dot{\lambda }}_{1}=K_{d}^{-1}F-K_{p}K_{d}^{-1}\Delta x+{ẋ}_{\text{d}}-J\left(\theta \right)\dot{\theta }, \end{array}$$

(15c) $$\begin{array}{r} \epsilon {\dot{\lambda }}_{2}=-\lambda_{2}+{\left(\lambda_{2}+J_{o}\dot{\theta }-B\right)}^{+}, \end{array}$$

with ϵ being a positive constant scaling the convergence of Equation (15).

The proposed RNN based algorithm is shown in Algorithm 1. Based on escape velocity method, the convex set of joint speed can be obtained based on the positive constant α and physical constraints θ^−^, θ^+^, ${\dot{\theta }}^{-}$, ${\dot{\theta }}^{-}$. After initializing state variables λ_1_ and λ_2_, the reference velocity can be obtained based on the desired command and actual responses according to Equation (4). then the output of RNN (which is also the control command) can be calculated based on Equation (15a), at the same time, both λ_1_ and λ_2_ can be updated according to Equations (15b) and (15c).

**Algorithm 1:** Collision-Free position-force controller based on RNN.

**Input:** Positive control gains α, ϵ, and interaction coefficients *K*_*p*_, *K*_*d*_. Initial states $\dot{q}\left(0\right)=0$, *q*(0), desired path *x*_d_(*t*), ẋ_d_(*t*) and operation force *F*_d_(*t*), task duration *T*_*e*_, feedback of end effector's coordination *x*(*t*) and contact force *F*, joint angles θ, Jacobian matrix *J*(θ), information of the obstacles *B*_*j*_ and Ḃ_*j*_ = 1, ⋯ , *b*. Location of key points *A*_*i*_, *i* = 1, ⋯ , *a* on the robot, and the corresponding Jacobian matrices *J*_*Ai*_. Physical limitations θ^−^, θ^+^, ${\dot{\theta }}^{-}$, ${\dot{\theta }}^{+}$. Safety distance *d*.
**Output:** To achieve position-force control without colliding with obstacles
1. Initialize λ_1_ = 0, λ_2_ = 0.%Joint velocity command *u*.
2. *x*, *q*, *F*, $\dot{\theta }$ ← Sensor readings
3. Calculate *x*_*Ai*_, ẋ_*Ai*_ and *J*_*Ai*_ by Equation (7)
4. Calculate matrices *J*_*o*_, *B* by Equation (11d)
5. Update upper and lower bounds of joint velocities by Equation (11c)
6. Update output of RNN (joint velocity) by $\ddot{\theta }$ using Equation (15a)
7. Update λ_1_ by ${\dot{\lambda }}_{1}$ using Equation (15b)
8. Update λ_2_ by ${\dot{\lambda }}_{2}$ using Equation (15c)
**Until**(*t* > *T*_*e*_)

In real applications, the nonlinear system can be hardly approximated completely. Therefore, the approximate error is inevitable, which would influence the performance of the proposed controller. However, the approximate error is a small value of higher order, and the influence can be suppressed based on the negative feedback scheme in the outer-loop, as shown in Equation (4).

*Remark 3*. The output dynamics of the proposed RNN is given in Equation (15a), in which the projection operator *P*_Ω_(•) plays an important rule in handling physical constraints (Equation 11c), the updating of $\dot{\theta }$ depends on three parts: the first part $-\dot{\theta }/||\dot{\theta }{||}_{2}^{2}$ in used to optimize the objective function $||\dot{\theta }{||}_{2}^{2}$, and the second item $J^{\text{T}}\lambda_{1}$ guarantees the equality constraint (Equation 11b) by adjusting the dual state variable λ_1_ according to Equation (15b), and the last item $-J_{o}^{\text{T}}\lambda_{2}$ ensures the inequality constraint (Equation 11d). The RNN consists of three kinds of nodes, namely, $\ddot{\theta }$, λ_1_ and λ_2_, with the number of neurons being *n* + *ab* + *m*.

It is remarkable that the proposed controller is based on the information of system models such as *J*, *J*_*o*_, *K*_*p*_, etc., which is helpful to reduce computational cost. As to the constraint-optimization problem (Equation 11), the main challenge is to solve it in real-time, since the parameters in constraints (Equations 11b, 11d) are time varying. From Equation (15), the control effort is obtained by calculating its updating law, which is based on the historical data and model information, i.e., it is no longer necessary to solve the solution of Equation (11) as every step, and the computational cost is thus reduced. In the following section, we will also show the convergence of the RNN based controller.

In this paper, we mainly concern the obstacle avoidance problem in force control tasks. It is notable that force control is mainly based on the idea of impedance control theory, which is similar to existing methods in Huang et al. (2019), Zhang and Xia (2019). The main challenge of the proposed control scheme lies in the limitation of sampling ability of cameras, which are used to capture the obstacles. To handle the measurement noise or disturbances, a larger safety distance *d* can be introduced to ensure the performance of obstacle avoidance.

### 3.2. Stability Analysis

*Lemma 1:* (Convergence for a class of neural networks) (Gao, 2003) A dynamic neural network is said to converge to its equilibrium point if it satisfies:

(16) $$\begin{array}{r} \kappa ẋ=-ẋ+P_{S}\left(x-\varrho F\left(x\right)\right), \end{array}$$

where κ > 0 and ϱ > 0 are constant parameters, and *P*_*S*_ = argmin_*y*∈*S*_||*y* − *x*|| is a projection operator to closed set *S*.

*Definition 1:* For a given function *F*(•) which is continuously differentiable, with its gradient defined as ∇*F*, if ∇*F* + ∇*F*^T^ is positive semi-definite, *F*(•) is called a monotone function.

About the stability of the closed-loop system, we offer the following theorem.

*Theorem 1:* Given the collision-free position-force controller based on a recurrent neural network, the RNN will converge to the inherent solution (optimal solution) of Equation (11), and the stability of the closed-loop system is also ensured.

*Proof:* Define a vector ξ as $\xi =\left[\dot{\theta };\lambda_{1};\lambda_{2}\right]\in {\mathbb{R}}^{n+m+ab}$, according to Equation (15), the time derivative of ξ satisfies:

(17) $$\begin{array}{r} \epsilon \dot{\xi }=-\xi +P_{\bar{\Omega }}\left[\xi -F\left(\xi \right)\right], \end{array}$$

in which ϵ > 0, and $F\left(\xi \right)={\left[F_{1}\left(\xi \right),F_{2}\left(\xi \right),F_{3}\left(\xi \right)\right]}^{\text{T}}$, where $F_{1}=\dot{\theta }/||\dot{\theta }{||}_{2}^{2}-J^{\text{T}}\lambda_{1}+J_{o}^{\text{T}}\lambda_{2}$, $F_{2}=J\dot{\theta }-K_{d}^{-1}F+K_{p}K_{d}^{-1}\Delta x-{ẋ}_{\text{d}}$, $F_{3}=-J_{o}\dot{\theta }+B$. By calculating the gradient of *F*(ξ), we have:

(18) $$\begin{array}{r} \nabla F\left(\xi \right)=\left[\begin{array}{ccc} I/||\dot{\theta }{||}_{2}^{2} & -J^{\text{T}} & J_{o}^{\text{T}}\\ J & 0 & 0\\ -J_{o}^{\text{T}} & 0 & 0 \end{array}\right]. \end{array}$$

It is obviously that ∇*F*(ξ) is positive definite. According to Definition 1, *F*(ξ) is a monotone function. From the description of (17), the projection operator *P*_*S*_ can be formulated as *P*_*S*_ = [*P*_Ω_; *P*_*R*_; *P*_Λ_], in which *P*_Ω_ is defined in (13a), *P*_*R*_ can be regarded as a projection operator of λ_1_ to *R*, with the upper and lower bounds being ±∞, and $P_{\Lambda }={\left(•\right)}^{+}$ is a special projection operator to closed set ${\mathbb{R}}_{+}^{ab}$. Therefore, *P*_*S*_ is a projection operator to closed set $\left[\Omega ;{\mathbb{R}}^{m};{\mathbb{R}}_{+}^{ab}\right]$. Based on Lemma 1, the proposed neural network (15) is stable and will globally converge to the optimal solution of (11).

Notable that the equality constraint 11(b) describes the impedance controller, and the convergence can be found in Na et al. (2015). Similarly, the establishment of inequality constraint enables obstacle avoidance during the whole process. The proof is completed.

*Remark 4*. It is remarkable that the original impedance controller described in 11(b) bears similar with traditional methods in Yang et al. (2018a) the main contribution of the proposed controller is that the controller can not only realize the force control, but also realize the obstacle avoidance, besides, the control strategy is capable of handling inequality constraints, including joint angles, and velocities.

## 4. Numerical Results

In this part, we carry out a series of numerical simulations on a planar 4-DOF robot, aiming at verifying the validity of the proposed control scheme. Firstly, a pure force control experiment is done to show the effectiveness of the force controller, and then the control scheme is further verified by examining the system response after introduction of obstacles. Then we check the control performance in more general situations, including model uncertainties and multiple obstacles.

### 4.1. Simulation Settings

**Figure 2:**
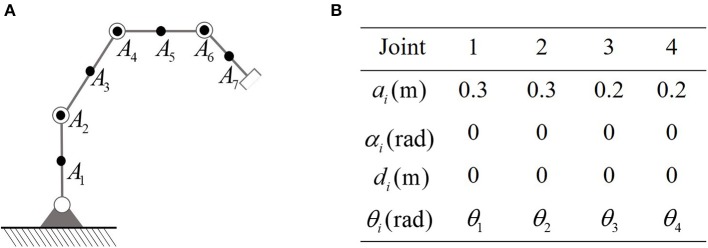
The robot to be simulated in this paper. **(A)** Is the physical structure and the location of key-points. **(B)** Is D-H parameters.

First of all, the planar robot used in the simulation is show in Figure 2. The D-H parameters are also listed in Figure 2B. It is remarkable that in force control tasks, the end-effector is required to keep in touch with workpieces, which makes it necessary to distinguish between the necessary contact and the unnecessary collisions. In this paper, the proposed controller is capable of handling this problem by selecting the key points properly. Therefore, the end-effector is not considered as a key point, to make it possible to contact with the obstacles (or external environment). In order to avoid obstacles, the set of key points of the robot is defined as *A*_1_, ⋯ , *A*_7_, in which *A*_1_, *A*_3_, *A*_5_, and *A*_7_ locate at the center of the links, and *A*_2_, *A*_4_, and *A*_6_ are defined to be at *J*2, *J*3, and *J*4, as shown in Figure 2A. The lower and upper bounds of joint angles and joint velocities are defined as $\theta_{i}^{-}=-3$rad, $\theta_{i}^{+}=3$rad, ${\dot{\theta }}_{i}^{-}=-1$rad/s, ${\dot{\theta }}_{i}^{+}=1$rad/s for *i* = 1…4, respectively. The safety margin is selected as 0.01 m. The coefficients describing the contact force are selected as *K*_*d*_ = 50, *K*_*p*_ = 5000. For simplicity, let $b_{0}=K_{d}^{-1}F-K_{p}K_{d}^{-1}\Delta x+{ẋ}_{\text{d}}$.

### 4.2. Force Control Without Obstacles

**Figure 3:**
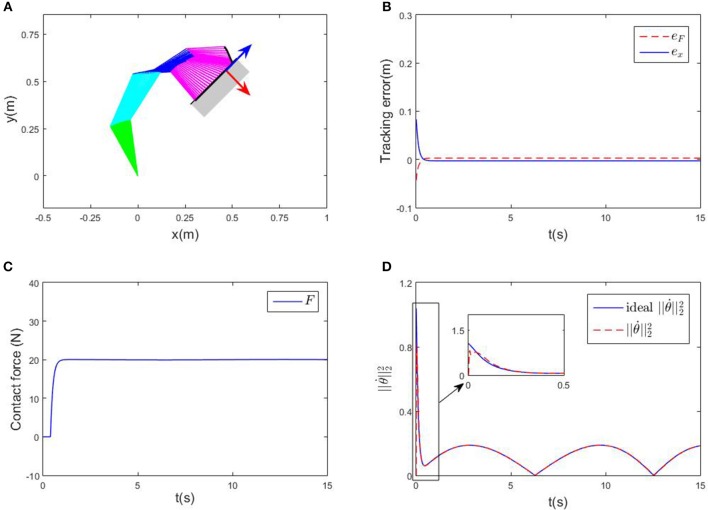
Numerical results of compliance control without obstacles. **(A)** Is the robot's tracking path and the corresponding joint configurations. **(B)** Is the profile of position error along the free-motion direction. **(C)** Is the profile of contact force. **(D)** Is the profile of $||\dot{\theta }{||}_{2}^{2}$.

First of all, an ideal case where there is no obstacles in the workspace is considered, and the parameters *K*_*d*_ and *K*_*p*_ are assumed to be known. The robot is wished to offer a constant contact force on a given plane. The contact force is set to be 20N, while the direction of contact force is aligned with the y-axis of the tool coordination system. In this example, the y-axis of is [1, −1]^T^ in the base coordination. The pre-defined path on the contact plane is *x*_d_ = [0.4+0.1*cos*(0.5*t*), 0.5+0.1*cos*(0.5*t*)]. The initial state of the robot system is set as $\theta_{0}={\left[1.57,-0.628,-0.524,-0.524\right]}^{\text{T}}$rad, ${\dot{\theta }}_{0}={\left[0,0,0,0\right]}^{\text{T}}$rad/s. The control gains of the proposed RNN controller are α = 8,ϵ = 0.02, respectively. Numerical results are shown in Figure 3. The tracking error along the contact plane is given in Figure 3B, the transient is about 1s. At the beginning stage, since the end-effector is not in contact with the surface, the contact force stays zero before 0.5s. As the end-effector approaches the surface, the contact force converges to 20N, showing the convergence of both positional and force errors. The Euclidean norm of joint velocities (which is also output of the established RNN) is shown in Figure 3D, $||\dot{\theta }||$ changes periodically, with the same cycle as the expected trajectory. The time history of the end-effector's motion trajectory and the corresponding joint configurations are shown in Figure 3A, in which the red arrow indicates the direction of the contact force, and the blue arrow shows the direction of the end-effector's free-motion. All in all, the proposed controller can achieve the position-force control precisely.

### 4.3. Force Control With Single Obstacles

In this section, a stick obstacle is introduced into the workspace, which is defined as *x* = −0.05 m. The initial states and expected values of *x*_d_, *F*_d_ are the same as section 4.2.

*Remark 5*. In Equation (10), we have shown the basic idea of calculating the distance between the robot and obstacles, i.e., by abstracting key points form the robot and obstacles, the distances can be the robot and obstacle can be described approximately at a set of point-to-point distances. In this example, the distance can be obtained in a simpler way. However, the obstacle avoidance strategy is essentially consistent with (Equation 10).

**Figure 4:**
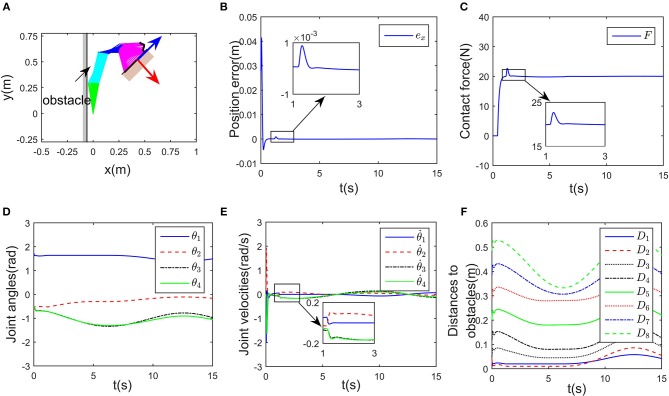
Control performance of the proposed controller while avoiding a wall obstacle. **(A)** Is the robot's tracking path and the corresponding joint configurations. **(B)** Is the profile of position error along the free-motion direction. **(C)** Is the profile of contact force. **(D)** Is the profile of joint angles. **(E)** Is the profile of joint velocities. **(F)** Is the profile of the closest distance to the obstacle of each key points *A*_*i*_, *i* = 1, ⋯ , 7.

**Figure 5:**
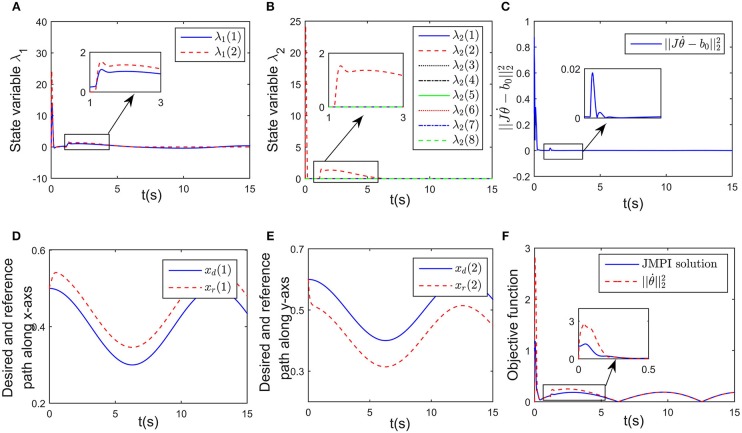
Simulation results of the established RNN while avoiding a wall obstacle. **(A)** Is the profile of λ_1_. **(B)** Is the profile of λ_2_. **(C)** Is the profile of $||J\dot{\theta }-b_{0}{||}_{2}^{2}$. **(D)** Is the profiles of the desired and reference trajectory along x-axis. **(E)** Is the profiles of the desired and reference trajectory along y-axis. **(F)** Is the profiles of the objective function of the proposed controller and JPMI based method.

Simulation results are given in Figures 4, 5. The output of RNN is shown in Figure 4E, when simulation begins, $\dot{\theta }$ reaches its maximum value, driving the end-effector to move toward the desired path. And then the robot slows down quickly (after *t*≈0.5s), the robot move smoothly, as a result, the position error successfully converges to 0, and simultaneously, the contact force converges to 20N. It is notable that at *t* = 1.2 s, the key point *A*_2_ of the robot gets close to the obstacle, as shown in Figure 4F. Based on the obstacle avoidance strategy (Equation 15c), the state variable λ_2_(2) becomes positive, and then the output of the RNN varies with λ_2_ (Figure 5B). Correspondingly, an error (about 1 × 10^−3^ m) occurs in the positional tracking, and so as the contact force (force error is about 2N). However, the RNN converges to the new equilibrium point(since the equilibrium point would change when the inequality constraint works), and both *e*_*x*_ and *e*_*f*_ converges to 0. By comparing Figures 3A, 4A, after introducing the obstacle, the robot is capable of adjusting its joint configuration to avoid the obstacle. The distances between the key points *A*_1_−*A*_7_ to the obstacle are shown in Figure 4D, a minimum value of about 0.01 m is ensured during the whole process. Using impedance model, the force control problem is transferred into a kinematic control one by modifying the reference speed (Equation 4). Consequently, the resulting trajectory *x*_*r*_ together with *x*_d_ are as shown in Figures 5D,E. As an important index in the proposed control scheme, the norm of joint speed $||\dot{\theta }{||}_{2}^{2}$ is wished as small as possible. Therefore, we introduce a comparative simulation, in which the solution is obtained based on pseudo-inverse of Jacobian matrix and physical limitations are not considered. Comparative curves of the objective functions are as shown in Figure 5F. The RNN based controller can optimize the objective function, it is remarkable that a difference appears at about *t* = 1.2−5 s, which is mainly caused by obstacle avoidance (which is not considered in JMPI based method). Since the output of RNN $\dot{\theta }$ is used to approximate the reference speed *b*_0_, the approximate error $||J\dot{\theta }-b_{0}{||}_{2}^{2}$ is shown in 55(C), demonstrating the effectiveness of the established RNN.

### 4.4. Force Control With Uncertain Parameters

**Figure 6:**
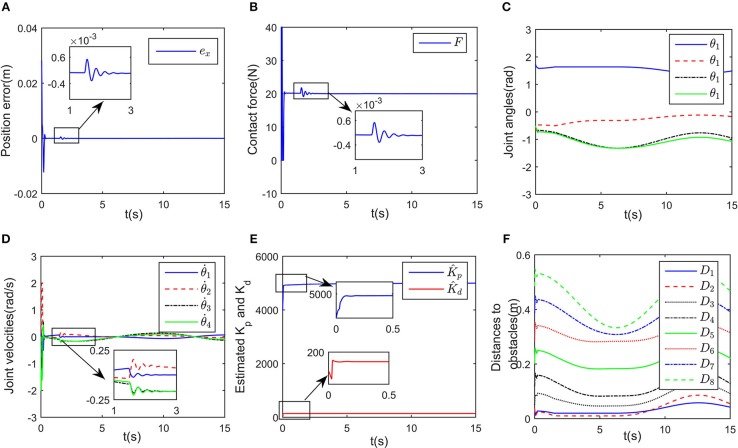
Control performance of the proposed controller while avoiding a wall obstacle with uncertain *K*_*p*_ and *K*_*d*_. **(A)** Is the robot's tracking path and the corresponding joint configurations. **(B)** Is the profile of position error along the free-motion direction. **(C)** Is the profile of contact force. **(D)** Is the profile of joint angles. **(E)** Is the profile of joint velocities. **(F)** Is the profile of the closest distance to the obstacle of each key points *A*_*i*_, *i* = 1, ⋯ , 7.

**Figure 7:**
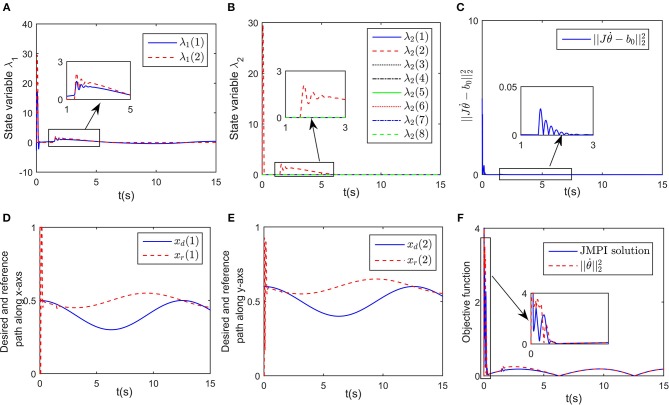
Simulation results of the established RNN while avoiding a wall obstacle with uncertain *K*_*p*_ and *K*_*d*_. **(A)** Is the profile of λ_1_. **(B)** Is the profile of λ_2_. **(C)** Is the profile of $||J\dot{\theta }-b_{0}{||}_{2}^{2}$. **(D)** Is the profiles of the desired and reference trajectory along x-axis. **(E)** Is the profiles of the desired and reference trajectory along y-axis. **(F)** Is the profiles of the objective function of the proposed controller and JPMI based method.

In this example, we check the control performance of the proposed control scheme in presence of model uncertainties. Similar with previous simulations, the initial states of the robot are also $\theta_{0}={\left[1.57,-0.628,-0.524,-0.524\right]}^{\text{T}}$rad, ${\dot{\theta }}_{0}={\left[0,0,0,0\right]}^{\text{T}}$rad/s. In real implementations, the interaction model is usually unknown, and the nominal values of *K*_*d*_ and *K*_*p*_ are not accurate. Without loss of generality, we select the nominal values of *K*_*d*_ and *K*_*p*_ as ${\hat{K}}_{d}=80$, ${\hat{K}}_{p}=4000$, respectively.In order to handle model uncertainties in the interaction coefficients, an extra node is introduced into (15). Then the modified RNN can be formulated as:

$$\begin{array}{l} \epsilon \ddot{\theta }=-\dot{\theta }+P_{\Omega }(\dot{\theta }-\dot{\theta }/||\dot{\theta }|{|}_{2}^{2}+J^{\text{T}}\lambda_{1}-J_{o}^{\text{T}}\lambda_{2}),\\ \epsilon {\dot{\lambda }}_{1}=K_{d}^{-1}F-K_{p}K_{d}^{-1}\Delta x+{\dot{x}}_{\text{d}}-J(\theta )\dot{\theta },\\ \epsilon {\dot{\lambda }}_{2}=-\lambda_{2}+{\left(\lambda_{2}+J_{o}\dot{\theta }-B\right)}^{+},\\ \;\dot{\hat{W}}=-K_{in}\eta {\left(F_{\text{d}}-F\right)}^{\text{T}}, \end{array}$$

in which *W* = [*K*_*p*_; *K*_*d*_], η = [*x* − *x*_d_; ẋ − ẋ_d_], and the positive coefficient *K*_*in*_ scaling the updating rate is defined as *K*_*in*_ = *diag*(500, 20). Simulation results are shown in Figures 6, 7. Although the exact values of *K*_*d*_ and *K*_*p*_ are unknown, the closed-loop system is still stable, which can be shown from the convergence of tracking error *e*_*x*_ and contact force *F* in Figures 6A,B. The change curves of joint angles and joint velocities with respect to time are shown in Figures 6C,D, in which the bounded-ness of joint angles and velocities are guaranteed. The observed interaction coefficients ${\hat{K}}_{d}$ and ${\hat{K}}_{p}$ are shown in Figure 6E, indicating that both ${\hat{K}}_{d}$ and ${\hat{K}}_{p}$ converge to their real values. Figure 7A shows the distances between the key points and the obstacle, it is obvious that all key points keep at a safe distance from the obstacle (the closest key point is *A*_2_). Euclidean norm of $b_{0}-J\dot{\theta }$ is illustrated in Figure 7C, despite fluctuation occurs at about *t* = 1.5 s, the proposed controller could handle model uncertainties. The impedance model based reference trajectory and the original desired trajectory are shown in Figures 7D,E. Although *x*_*r*_ and *x*_d_ are different, the tracking error *e*_*x*_ along the direction of free motion and force error *e*_*F*_ converges to zero, as shown in Figures 6A,B. The objective function $||\dot{\theta }{||}_{2}^{2}$ to be optimized is given in Figure 7F. the convergence of the established RNN is shown in Figure 7C, despite the uncertain parameters, using the adaptive updating law, the established RNN is capable of learning the optimal solution. The spikes is mainly because of the change of λ_2_ when obstacle avoidance scheme is activated.

### 4.5. Manipulation in Narrow Space

**Figure 8:**
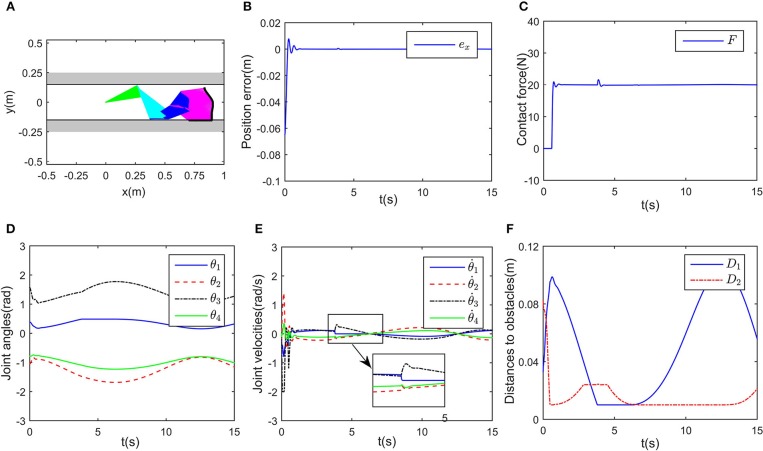
Control performance of the proposed controller in a narrow workspace. **(A)** Is the robot's tracking path and the corresponding joint configurations. **(B)** Is the profile of position error along the free-motion direction. **(C)** Is the profile of contact force. **(D)** Is the profile of joint angles. **(E)** Is the profile of joint velocities. **(F)** Is the profile of the closest distance to the obstacle of each key points *A*_*i*_, *i* = 1, ⋯ , 7.

**Figure 9:**
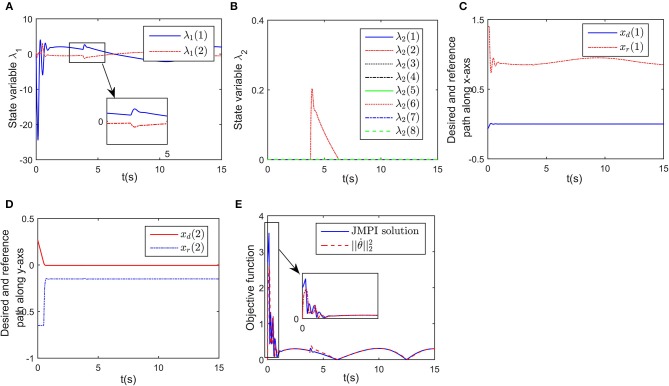
Simulation results of the established RNN in a narrow workspace. **(A)** Is the profile of λ_1_. **(B)** Is the profile of λ_2_. **(C)** Is the profiles of the desired and reference trajectory along x-axis. **(D)** Is the profiles of the desired and reference trajectory along y-axis. **(E)** Is the profiles of the objective function of the proposed controller and JPMI based method.

In this part, we discuss a more general case of motion-force control task, in which the workspace is defined in a limited narrow space. The robot is limited by two parallel lines, namely, *y*_1_ = 0.15 and *y*_2_ = −0.15 m. Considering the safety distance, all key points except *A*_8_ must satisfy the workspace description −0.14 ≤ *y* ≤ 0.14 m. The initial joint angles are set to be $\theta_{0}={\left[0.393,-1.05,1.57,-0.785\right]}^{\text{T}}$rad, and ${\dot{\theta }}_{0}={\left[0,0,0,0\right]}^{\text{T}}$rad/s. The desired path is selected as $x_{\text{d}}={\left[0.8+0.1cos\left(0.5t\right),-0.15\right]}^{\text{T}}$ m, and the expected contact force is *F*_d_ = 20N, with the direction vector being [0, −1]^T^. Simulation results are given in Figures 8, 9. When simulation begins, the initial position error is about 0.1 m, and the converges to zero, with the transient being about 0.5s. Simultaneously, the contact force also converges to 20N. In Figure 9A, minimum distances between the key points to *y*_1_ and *y*_2_ are represented by blue and red curves, respectively. The tracking trajectory and the corresponding joint configurations are shown in Figure 8A. During *t* = 1 − 1.5 s and *t* = 6 − 13 s, point *A*_2_ gets close to *y*_1_, during *t* = 4 − 7 s, *A*_4_ is close to *y*_2_. Remarkable that there exist fluctuations in positional and force errors at *t* = 1 s and *t* = 4 s (i.e., when *A*_2_ and *A*_4_ get close to the bounds), respectively. Similar to the previous simulations, the reference trajectories are given in Figures 7C,D, and the objective functions are shown in Figure 7E. Using the proposed RNN controller, the robot can realize both position and force control in limited narrow space.

### 4.6. Comparisons

In this part, comparisons among the proposed control scheme and existing methods are given to show the superiority of the RNN based strategy. The comparisons are shown in Table 1. In Guo and Zhang (2012), a RNN based controller is designed for redundant manipulators, both obstacle avoidance and physical constraints are considered. However, the controller only focus on kinematic control problem. In Nanayakkara et al. (2001) and Csiszar et al. (2011), force control together with obstacle avoidance are taken into account, but the physical constraints are ignored. Xu et al. (2019a) develop an adaptive admittance control strategy, which is capable of dealing with force control under model uncertainties, physical constraints and real-time optimization. It is remarkable that the proposed strategy focus on real-time obstacle avoidance in force control tasks, and the controller is capable of ensuring the boundedness of joint angles and velocities. At the same time, simulations have shown the potential of optimization ability of norm of joint speed.

**Table 1:** Comparisons among the proposed controller and existing methods.

**Method**	**Convergence**	**Real-time**	**Physical**	**Force control/**	**Collision**
		**optimization**	**constraints**	**kinematic control**	**free**
This paper	Yes	Yes	Considered	Force control	Yes
Guo and Zhang (2012)	Yes	Yes	Considered	kinematic control	Yes
Nanayakkara et al. (2001)	Yes	Yes	Ignored	Force control	Yes
Xu et al. (2019a)	Yes	Yes	Considered	Force control	No
Csiszar et al. (2011)	Yes	No	Ignored	Force control	Yes

## 5. Conclusions

In this paper, a novel collision-free compliance controller is constructed based on the idea of QP programming and neural networks. Different with existing methods, in this paper, the control problem is described from an optimization perspective, and the compliance control and collision avoidance are formulated as equality or inequality constraints. The physical constraints such as limitations of joint angles and velocities are also taken into consideration. Before ending this paper, it is worth pointing out that it is the first RNN based compliance control method, which considers collision avoidance problem in realtime, and also shows great potential in handling physical limitations. In this paper, simple numerical simulations in MATLAB are carried out to verify the efficiency of the proposed controller. In the future, we will check the control framework with different impedance models in physically realistic simulation environments, and then consider machine vision technology and system delay problem on physical experimental platforms.

## Data Availability

All datasets analyzed for this study are included in the manuscript and the supplementary files.

## Author Contributions

XZ designed the control strategy and writes the paper. ZX did the numerical experiments. SL revised the paper and proposed amendments.

### Conflict of Interest Statement

The authors declare that the research was conducted in the absence of any commercial or financial relationships that could be construed as a potential conflict of interest.
